# Traditional Oriental Medicines and Alzheimer’s Disease

**DOI:** 10.14336/AD.2018.0328

**Published:** 2019-04-01

**Authors:** Seong Gak Jeon, Eun Ji Song, Dongje Lee, Junyong Park, Yunkwon Nam, Jin-il Kim, Minho Moon

**Affiliations:** ^1^Department of Biochemistry, College of Medicine, Konyang University, Daejeon 35365, Republic of Korea; ^2^Center for Organic Devices and Advanced Materials, Kyungsung University, Busan 48434, Republic of Korea; ^3^Department of Nursing, College of Nursing, Jeju National University, Jeju-si 63243, Republic of Korea

**Keywords:** Alzheimer’s disease, Oriental herbal medicine, Herbal formulae, Dementia

## Abstract

Alzheimer’s disease (AD), which is the most major cause of dementia, is a progressive neurodegenerative disease that affects cognitive functions. Even though the prevalence of AD is continuously increasing, few drugs including cholinesterase inhibitors and N-methyl D-aspartate-receptor antagonists were approved to treat AD. Because the clinical trials of AD drugs with single targets, such as β-amyloid and tau, have failed, the development of multi-target drugs that ameliorate many of the symptoms of AD is needed. Thus, recent studies have investigated the effects and underlying mechanisms of herbal formulae consisting of various herb combinations used to treat AD. This review discusses the results of clinical and nonclinical studies of the therapeutic efficacy in AD and underlying mechanisms of the herbal formulae of traditional Oriental medicines and bioactive compounds of medicinal plants.

Alzheimer’s disease (AD), the most major cause of dementia, is a progressive neurodegenerative disease involving characteristic pathologic changes, including the accumulation of β-amyloid (Aβ) and neurofibrillary tangles (NFT) [1-4]. AD results in devastating health-related consequences, such as economic burden, decreased quality of life for the patients and their caregivers and social problems [5-7]. Because the number of AD patients continues to grow, many pharmacological and nonpharmacological interventions to improve the symptoms as well as ameliorate the pathologic changes of AD have been investigated. These interventions directly target acetylcholinesterase (AChE), Aβ peptide, or tau proteins [8-12] and/or modulate the signaling pathways underlying Aβ oligomer formation, tau hyperphosphorylation, or other AD-related pathologies, such as inflammation, oxidative stress and impaired adult neurogenesis [12-16]. Currently, only symptom-relieving drugs, such as AChE inhibitors (donepezil, galantamine and rivastigmine) and N-methyl D-aspartate (NMDA) receptor blocker (memantine), have been approved for use by the Food and Drug Administration (FDA) [17]. Moreover, despite ongoing preclinical/clinical trials, no cure for AD has been developed [4].

Based on the lack of disease-modifying drugs for AD, traditional medicines, such as natural herbal products, have been used to enhance treatment of the symptoms and pathologic processes of AD. Traditional Oriental medicines (TOMs), including herbs, from East Asian countries have long been used to treat the symptoms of dementia [18]. Recent studies have revealed that the pharmacologic compounds in TOMs have potent therapeutic effects in AD [18-20]. However, these studies have mainly focused on TOM compounds or ingredients. Indeed, the formulae of traditional medicines from East Asian countries, including China, Korea and Japan, commonly consist of multiple herbs [21]. Moreover, the formulae of traditional medicines from East Asian countries consisting of the same herbs can differ considerably [22]. Thus, the effects of mixtures of standardized formulae of TOMs need to be evaluated.

Nonetheless, few reviews have been conducted on studies of the clinical effects of TOMs on AD. Such reviews would provide a better understanding of possible strategies in the treatment of AD. Reportedly, there are abundant number of clinical or non-clinical studies revealing the beneficial effects of TOMs. In particular, reviewing TOMs prescribed for patients with cognitive impairments or aging-related symptoms in clinical settings such as *Yi gan san* (YGS) [23], *Ba wei di huang wan* (BWDHW) [24], *Jiawei wen dan tang* [25], *Danggui shaoyao san* [26], *Huanglian jiedu tang* (HLJDT) [27] might be more advantageous for finding potentials of TOMs on AD treatment. This review focuses on the recent findings of clinical and nonclinical studies of the mechanisms of action and therapeutic effects of five TOM formulae commonly used in the clinical treatment of patients with cognitive impairments and related symptoms.

## 1. Overview of TOMs and AD

Herbs, which have been used in Oriental medicine in East Asian countries for over 2,000 years, are currently being investigated scientifically. Treatment strategies using TOMs are of interest for many researchers because pharmacological treatments of AD have not yet been successful. A recent study performed a systematic literature review of 731 citations in 127 premodern Chinese medical books and identified 31 herbs used to treat memory disorders [28]. Another study reviewed the composition and underlying mechanisms of several natural active ingredients, including flavonoids, alkaloids and polysaccharides, that were isolated from Chinese herbal medicine and that have ameliorating effects on patients with AD [29]. An investigation of the therapeutic effects of herbal medicine and acupuncture on senile dementia found that the combined use of Chinese herbal medicine and acupuncture had potential efficacy against senile dementia [30]. Moreover, several studies have concluded that Oriental herbal medicines have positive effects on cognitive functions, including learning and memory, in patients with AD and vascular dementia [31-33]. Recent studies focused on the effectiveness of polysaccharides derived from TOMs and single plants that traditionally used for AD-like symptoms or not for AD-like symptoms have reviewed their various effects and mechanisms [34, 35]. Moreover, one meta-analysis study has concluded that profitable effects of TOMs were not different as compared to galantamine, rivastigmine and memantine [36]. The results of these studies offer new perspectives on the therapeutic use of TOMs in the treatment of AD.

Although few studies have reviewed the evidence for the effects of TOMs consisting of multiple herbs, combined forms of TOMs have often been prescribed and used in practice [28, 37]. Multiple lines of evidence suggest not only the effects of single herbs on AD but also the effects and underlying mechanisms of herbal formulae on AD. Thus, in this review, we discuss the effects and mechanisms of formulae used in the treatment of AD and suggest new perspectives of the use of alternative and complementary medicine to treat patients with AD.

## 2. TOM formulae used in the treatment of AD in East Asian countries

Table 1 presents a summary of the efficacy and herb constituents of five TOM formulae used in the treatment of AD.

### 1) Effects of YGS on AD

YGS (*Yi gan San* in Chinese, *Ukgansan* in Korean and *Yokukansan* in Japanese), which was described in a Chinese pediatric medical book (*Bao ying cuo yao* ) published in 1,555, was originally developed to treat agitation and restlessness in children [38]. It consists of *Atractylodes lancea* (AL) Thunb. DC. rhizome, *Wolfiporia extensa* Peck Ginns sclerotium, *Angelica acutiloba* (AA) Siebold & Zucc. Kitag. root, *Cnidium officinale* Makino rhizome, *Uncaria rhynchophylla* (UR) Miq. Jacks. thorn, *Bupleurum falcatum* (BF) L. root and *Glycyrrhiza uralensis* (GU) Fisch. root [39-41].

After a randomized clinical study reported attenuating effects of YGS on the behavioral and psychological symptoms of dementia (BPSD) in patients with dementia [42], preclinical and clinical trials have reported direct evidence of the positive pharmacological effects of YGS in AD.

**Table 1:**
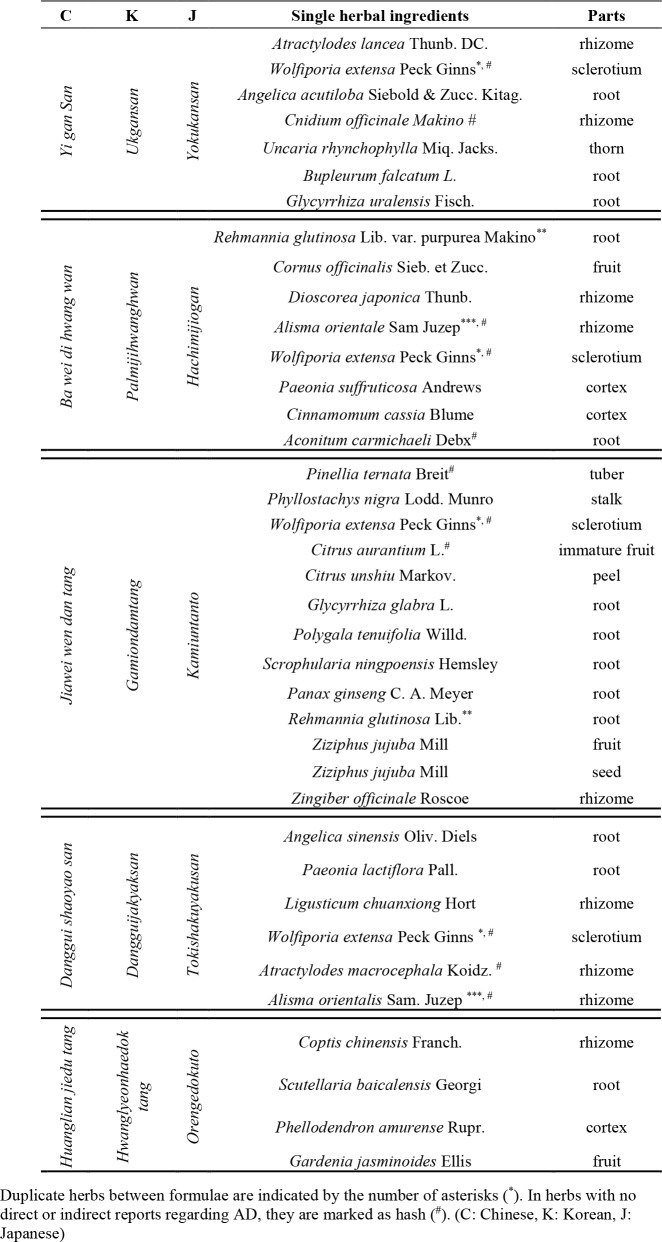
Composition of five herbal formulae for the treatment of AD.

#### (1) In vivo and in vitro studies of YGS

Tg2576 mice, an animal model of AD, that were administered YGS showed improved performances on elevated plus-maze and Morris water-maze (MWM) tests, which suggested that YGS enhanced learning and memory functions and decreased anxiety and locomotor hyperactivity in an open-field test [43]. Interestingly, following reports of the inhibitory effects on Aβ aggregation of the thorns and stems of UR, which is a YGS ingredient [44], YGS was shown to suppress Aβ aggregation *in vitro* , while treatment with YGS or UR thorn was shown to prevent abnormal social interaction and memory disturbance in amyloid precursor protein transgenic mice [45]. However, the inhibitory effects of YGS on Aβ aggregation should be further examined because conflicting results of YGS treatment (1.0% for 10 months) on Aβ accumulation in mice have been reported [43]. One study demonstrating cholinesterase-inhibiting effects and cognitive-enhancing effects of YGS in early AD model rats has suggested the therapeutic efficacy of YGS in the treatment of the cognitive impairments of patients with AD. The cognitive-enhancing effects of YGS are mediated by increased levels of acetylcholine (ACh) and dynamin-1, a protein involved in synaptic vesicle recycling at neuronal synapses [46]. In addition to the effects of YGS on cholinergic neurotransmission, isoliquiritigenin, a component of GU root, inhibited NMDA receptors in rat cultured cortical neurons, which would ameliorate the negative effects of excessive glutamatergic transmission, a key process in cognitive dysfunction in AD [47].

**Table 2:** The efficacy and therapeutic mechanisms of single herbs constituting YGS on AD.

	Single herbs	Bioactive materials	Efficacy and mechanisms	Ref.
Y G S	*Atractylodes lancea*		Antioxidative effect	[54]
		β-eudesmol	Inducing neurite outgrowth via MAPK activation, Increasing intracellular Ca^2+^ level induced by PI-PLC activation	[56]
	*Angelica acutiloba*		Ameliorating repeated cerebral ischemia-induced memory impairment	[57]
			Increasing Ach levels and decreasing neuronal apoptosis in the dorsal hippocampus	
			Alleviating cognitive impairment induced by scopolamine	[58]
	*Uncaria rhynchophylla*		Inhibiting Aβ aggregation	[44]
			Suppressing the level of lipid peroxides	[59,60]
			Improving cognitive function and decreasing AChE activity Antioxidative effect	[61]
		Rhynchophylline	Protecting Aβ-induced cytotoxicity via inhibitiion of intracellular Ca^2+^ overloading and tau hyperphosphorylation	[62]
		Isorhynchophylline		
		Geissoschizine	Non-competitive inhibition against AChE	[63]
		Uncarinic Acid C	A specific inhibitor for the Aβ_42_ aggregation in nucleation phase	[64]
	*Bupleurum falcatum*			
		Saikosaponin C	Suppressing the release of Aβ_1-40_ Aβ_1-42_ in various neuronal models Increasing neurite outghrowth by inhibiting tau hyperphosphorylation	[65]
	*Glycyrrhiza uralensis*		Alleviating cognitive impairment induced by Aβ administration	[66]
			Reducing activity of AChE and catalase in the brain	
		Isoliquiritigenin	Inhibiting Aβ aggregation and reducing Aβ toxicity	[67]
	*Cnidium officinale*		No direct reports regarding AD	
	*Wolfiporia extensa*		No direct reports regarding AD	

Aβ: amyloid beta, Ach: acetylcholine, AChE: acetylcholinesterase, AD: Alzheimer’s disease, Akt: protein kinase B (PKB), i.c.v: Intracerebroventricular, MAPK: mitogen-activated protein kinase, PI3K: phosphoinositide 3-kinase, PI-PLC: phosphoinositide-specific phospholipase C.

#### (2) Clinical trials of YGS

Randomized single-blinded clinical studies found that 4 weeks of YGS treatment ameliorated the BPSD in patients with AD and dementia with Lewy bodies [48] and 12 weeks of YGS treatment improved BPSD in patients with AD [49]. Additionally, a nonblinded clinical trial reported that 4 weeks of YGS administration significantly improved the BPSD in patients with AD who were being treated with regular AD treatments [50].

These attenuating effects of YGS on the BPSD in patients with dementia may be associated with the stimulation or inhibition of serotonergic, glutamatergic, cholinergic, dopaminergic and GABAergic neuro-transmission [38]. Notably, several studies that have examined the involvement of YGS or its constituent herb in the interaction on neurotransmitter receptors involved in AD symptoms suggest that YGS may be applicable in the treatment of AD [51-53]. Thus, the results of these studies suggest that the additional use of YGS in the treatment of AD will be beneficial.

#### (3) Efficacy and mechanisms of action in AD pathology of each herb in YGS

Efficacy of single herbs of BWDHW is described on Table 2.

##### a) AL rhizome

AL root extract is known to be an antioxidant [54]. As the process of neurodegeneration in AD patients is influenced by increased oxidative stress [55], antioxidative features of root of AL might be involve in beneficial effects of YGS. More on antioxidative effects, β-eudesmol, one of the major constituents of the root extract of AL, induced neurite outgrowth from rat pheochromocytoma cells (PC-12) cells through mitogen-activated protein kinase (MAPK) activation as well as intracellular Ca^2+^ level increase induced by phosphoinositide-specific phospholipase C activation [56].

##### b) AA root

Although there were no direct studies regarding AD, one study has reported that treatment of AA root extract ameliorated repeated cerebral ischemia-induced memory impairment by increasing ACh levels in the dorsal hippocampus. In addition to the cognitive enhancing effects, AA root extract also prevented the neuronal apoptosis in the hippocampus [57]. Another study has shown that isolated fractions from AA root (extracted by butanol) improved scopolamine-induced memory impairment of rats [58].

##### c) UR thorn

UR thorn extract inhibits Aβ aggregation and acts as an antioxidant by suppressing lipid peroxides [44, 59, 60]. MWM and open-field test results in a mouse model have shown that UR administration improves D-galactose-induced cognitive impairments and decreased AChE activity [61]. In particular, the alkaloids rhynchophylline and isorhynchophylline isolated from UR have been shown to protect against Aβ-induced cytotoxicity by inhibiting intracellular Ca^2+^ overloading and tau protein hyperphosphorylation [62]. In addition, geissoschizine methyl ether isolated from UR exhibited reversible and noncompetitive inhibition against AChE [63], and uncarinic acid C isolated from UR specifically inhibited Aβ_42_ aggregation in the nucleation phase [64].

##### d) BF root

Saikosaponin C, one of oleanane-type triterpenes from BF, has shown to suppress both release of Aβ_1-40_, Aβ_1-42_ in various neuronal models and increase neurite outgrowth by inhibiting abnormal tau phosphorylation in PC12 cells as well. However, activity and expression of β-site amyloid precursor protein cleaving enzyme 1 (BACE1), which is very involved in Aβ generation, did not changed after saikosaponin C treatment [65].

##### e) GU root

The results of passive avoidance tests (PATs) and MWM tests have demonstrated that GU water extract administration alleviated cognitive impairments induced by Aβ administration in mice. In addition, AChE and catalase activities were significantly reduced in the brain [66]. Moreove, isoliquiritigenin, one major component of GU, not only prevents Aβ-aggregation but also inhibits acute Aβ toxicity in human Aβ overexpression in *Caenorhabditis elegans* [67].

### 2) Effects of BWDHW on AD

BWDHW (*Ba wei di huang wan* in Chinese, *Palmijihwanghwan* in Korean and *Hachimijiogan* in Japanese), is composed of the following eight herbs: *Rehmannia glutinosa* (RG) Libosch. *var. purpurea* Makino root, *Cornus officinalis* (COF) Sieb. et Zucc. fruit, *Dioscorea japonica* (DJ) Thunb. rhizome, *Alisma orientale* Sam Juzep. rhizome, *Wolfiporia extensa* Peck Ginns sclerotium, *Paeonia suffruticosa* (PS) Andrews cortex, *Cinnamomum cassia* (CC) Blume cortex and *Aconitum carmichaeli* Debx root [68].

**Table 3:** The efficacy and therapeutic mechanisms of single herbs constituting BWDHW on AD.

	Single herbs	Bioactive materials	Efficacy and mechanisms	Ref.
B W D H W	*Rehmannia glutinosa*		Improving scopolamine-induced cognitive impairment and cholinergic dysfunctions with decreasing TNF-α and IL-1β mRNA expression	[75]
			Increasing the gene expression of GDNF in astroglial cells via ERK1/2 and cPKC	[76]
		Catalpol	Increasing ChAT and BDNF levels	[73]
			Improvement of Aβ-induced memory and learning impariment via reducing Aβ and regulating ROS related enzymes	[74]
	*Cornus officinalis*			
		Loganin	Improving scopolamine-induced memory impairment and significantly inhibit AChE activity	[77]
		p-Coumaric acid	Inhibiting BACE1 via bind to the β-secretase subsite or to another regulatory site	[78]
		Gallic acid	Inhibiting BACE1 activity	
		Ursolic acid		
		1,2,3,6-tetra-O-galloyl-β-D-glucose	Inhibiting BACE1 via interaction with both the peripheral anionic sites and the catalytic active sites	[79]
		Tellimagrandin II	Inhibiting ChEs via interaction with both the peripheral anionic sites and the catalytic active sites	
	*Dioscorea japonica*			
		Coreajaponin B	Upregulating NGF without cell toxicity	[80]
	*Paeonia suffruticosa*			
		1,2,3,4,6-penta-O-galloyl-β-D-glucopyranose	Inhibiting the Aβ-aggregation, destabilizing the pre-formed Aβ fibrils Alleviating long-term memory impairment in Tg2576 mice	[81]
		Paeonol	Improving the impaired learning behavior induced by Aβ intra-hippocampal injection Suppressing apoptosis via upregulation of cytochrome oxidase and α-actin	[82]
	*Cinnamomum cassia*		Improvement of cognitive function and glucose homeostasis damaged by Aβ accumulation via enhancing insulin signaling and suppressing TNF-α and iNOS	[83]
	*Alisma orientale*		No direct reports regarding AD	
	*Aconitum carmichaeli*		No direct reports regarding AD	
	*Wolfiporia extensa*		No direct reports regarding AD	

Aβ: amyloid beta, AChE: acetylcholinesterase, AD: Alzheimer’s disease, BACE1: beta- site amyloid precursor protein cleaving enzyme 1, BDNF: brain-derived neurotrophic factor, ChAT: choline acetyltransferase, ChEs: cholinesterases, cPKC: conventional protein kinase C, ERK1/2: extracellular signal-regulated kinases. 1/2, GDNF: glial cell line-derived neurotrophic factor, IL-1β: interleukin 1 beta, iNOS: inducible nitric oxide synthase, NGF: nerve growth factor, ROS: reactive oxygen species, Tg2576: mouse model of AD with APP Swedish mutation (KM670/671NL), TNF-α: tumor necrosis factor alpha.

#### a) In vivo and in vitro studies of BWDHW

BWDHW treatment (100 µg for 7 days) stimulated neurite outgrowth from PC12m3 cells under NGF [69]. In addition, the results of animal experiments provide more evidence for the effects of BWDHW on cognitive function. In rats with scopolamine-induced memory disturbance, the administration of BWDHW significantly increased the number of correct choices and decreased the number of error choices made in a radial maze test [70]. Moreover, BWDHW (0.5 g/kg) administration prolonged the step-through latencies that were shortened in the PAT after amnesia was induced by scopolamine, cycloheximide and cerebral ischemia [71]. In addition, BWDHW increased the levels of ACh, choline acetyltransferase (ChAT) activity in the frontal cortex of normal rats [70]. Thus, the underlying mechanisms of the cognitive-enhancing effects of BWDHW are, in part, associated with effects that preserve ACh content and modulate AChE and ChAT activity. Because most FDA-approved drugs for AD are AChE inhibitors, BWDHW might serve as a therapeutic agent in the treatment of cognitive dysfunction in AD.

#### b) Clinical trials of BWDHW

A randomized clinical trial has shown that BWDHW treatment is beneficial in elderly patients with mild to severe dementia. An experimental group treated with 2 g of BWDHW 3 times a day for 8 weeks showed significantly increased scores on the Mini-Mental State Examination (MMSE) and Barthel Index [24]. In addition, patients with multiple cerebral infarctions treated with BWDHW for 8 weeks exhibited improved cognitive function and significantly increased cerebral blood flow (CBF) in the whole brain and multiple regions (temporal lobe, Broca’s area and thalamus) [72]. Although these studies examined patients with various types of dementia, the results suggest possible benefits of the use of BWDHW in AD.

#### (1) Efficacy and mechanisms of action in AD pathology of each herb in BWDHW

Efficacy of single herbs of BWDHW is described on Table 3.

##### a) RG root

Direct evidence on the efficacy of RG root in AD treatment has not been reported. However, RG-derived catalpol, an iridoid glycoside from RG, improved the learning and memory impairments exhibited in Aβ-induced neurodegenerative mouse models by decreasing Aβ, regulating radical oxygen species-related enzymes and increasing brain-derived neurotrophic factor (BDNF) level [73, 74]. In addition, MWM test and PATs results have demonstrated that the administration of steamed RG root improved scopolamine-induced cognitive dysfunction and cholinergic immunoreactivity impairments through the decreased expression of tumor necrosis factor-α (TNF-α) and interleukin-1β (IL-1β) mRNA [75]. Moreover, RG increased the gene expression of glial cell line-derived neurotrophic factor (GDNF) in astroglial cells through phosphorylation of extracellular signal-regulated kinase 1/2 (ERK1/2) independently of cyclic adenosine monophosphate (cAMP)-dependent protein kinase C pathways [76].

##### b) COF fruit

The methanolic extract of COF and its major component, loganin, have been reported to improve scopolamine-induced memory impairments and significantly inhibit AChE activity in mice [77]. In addition, p-coumaric acid, gallic acid and ursolic acid derived from the ethyl acetate fraction of COF inhibited BACE1 activity. In particular, p-coumaric acid showed to inhibit BACE1 activity dose-dependently via binding to the β-secretase subsite or to another regulatory site [78]. Moreover, tellimagrandin II and 1,2,3,6-tetra-O-galloyl-b-D-glucose, the constituents of COF, showed inhibiting effects on AChE and BACE1 respectively via interaction with both the peripheral anionic sites and the catalytic active sites of each enzyme [79].

##### c) DJ rhizome

Direct evidence for the efficacy of the DJ rhizome in AD treatment has not been reported. However, coreajaponin B, which is obtained by purifying a DJ extract from 50% aqueous alcohol through multiple chromatographic steps, has shown to up-regulate nerve growth factor (NGF) without cell toxicity in a C6 rat glioma cell line [80].

##### d) PS cortex

PS cortex and its active ingredient 1,2,3,4,6-penta-O-galloyl-β-D-glucopyranose has been shown to inhibit Aβ_1-40, 1-42_ fibril formation, diminish pre-formed Aβ fibrils and alleviate long-term memory impairments in Tg2576 mice as well [81]. In addition, paeonol (2'-hydroxy-4'-methoxyacetophenone;1-(2-hydroxy-4-methoxyphenyl)ethan-1-one), one of the active ingredients of PS, improved learning behavior impairments induced by Aβ_1-42_ intrahippocampal injections in rat models via the suppression of apoptosis with elevated levels of cytochrome oxidase 1 in the hippocampus, cortex and vascular α-actin [82].

##### e) CC cortex

CC cortex has been shown to improve cognitive function and glucose homeostasis damaged by hippocampal CA1 infusion of Aβ_25-35_ in rats via reducing Aβ deposition, enhancing insulin signaling and suppressing inflammatory mediator such as TNF-α and inducible nitric oxide synthase (iNOS) [83].

### 3) Effects of JWWDT on AD

JWWDT (*Jiawei wen dan tang* in Chinese, *Gamiondamtang* in Korean and *Kamiuntanto* in Japanese) is traditionally used to treat neurosis and insomnia [84, 85]. JWWDT consists of the following 13 herbs: *Pinellia ternata* Breit. tuber, *Phyllostachys nigra* (PN) Lodd. Munro stalk, *Wolfiporia extensa* Peck Ginns sclerotium, *Citrus aurantium* L. immature fruit, *Citrus unshiu* (CU) Markov. peel, *Glycyrrhiza glabra* (GG) L. root, *Polygala tenuifolia* (PT) Willd. root, *Scrophularia ningpoensis* (SN) Hemsley root, *Panax ginseng* (PG) C.A. Meyer root, RG root, *Ziziphus jujuba* (ZJ) Mill. fruit, ZJ seed and *Zingiber officinale* (ZO) Roscoe rhizome [84]. JWWDT has been reported to improve the cognitive symptoms of AD.

**Table 4:** The efficacy and therapeutic mechanisms of single herbs constituting JWWDT on AD.

	Single herbs	Bioactive materials	Efficacy and mechanisms	Ref.
J W W D T	*Phyllostachys nigra*		Protecting against neuronal cytotoxicity by Aβ_25-35_ by decreasing elevated levels of Ca^2+^, glutamate release and reactive oxygen species (ROS) generation	[91]
	*Citrus unshiu*			
		Nobiletin	Protecting H2O2-induced cytotoxicity via suppression of activation of JNK, p38 and expression of Bax and Caspase 3	[92]
	*Glycyrrhiza glabra*		Enhancing the spatial memory impairment induced by scopolamine or diazepam via antioxidant and anti-inflammatory activities	[93,94]
			Reducing AChE activity	[96]
		2,2',4'-trihydroxychalcone	Mitigating the memory impairment in the APP-PS1 double transgenic mouse model via reducing the production of Aβ by specific, non-competitive inhibition of BACE1	[95]
	*Polygala tenuifolia*		Increasing axonal length and decreasing number of damaged neurons in Aβ_25-35_-treated neurons	[97]
			Improving the scopolamine-induced cognitive decline	[98]
			Inhibiting AChE activity non-competitive and dose-dependent manner	
			Protecting the cell death induced by Aβ, C-terminal fragment of APP and glutamate	
	*Scrophularia ningpoensis*			
		Harpagoside	Improvement of neurite outgrowth and ChAT (+) neurons reduced by Aβ_1-40_	[99]
			Alleviating the cognitive decline and neurodegenerative changes induced by Aβ via increasing BDNF and up-regulating MAPK/PI3K pathway	
	*Panax ginseng*		Promoting the cognitive function by reducing levels of Aβ_1-42_ and phosphorylated tau contents via activation of PI3K/Akt signaling pathway	[100]
			Alleviating cognitive impairment in Tg mAPP mice by decreasing Aβ_1-40_, Aβ_1-42_ levels, γ-secretase activity and increasing PKA/CREB signaling pathway	[101]
			Increased ADAS and MMSE scores in AD patients	[102]
	*Ziziphus jujuba*			
	(fruit)	Shakin-Z	Antioxidative effect / inhibiting the BChE and AChE	[103]
	(seed)	Spinosin	Alleviating AβO-induced cognitive decline / inhibiting the activation of microglia and ChAT	[104]
			Alleviating the scopolamine-induced memory impairment / increasing phosphorylation of ERK and CREB in the hippocampus	[105]
			Enhancing cognitive functions via upregulating adult hippocampal neurogenesis and activating ERK/CREB and BDNF pathway	[106]
	*Zingiber officinale*		Inhibiting the Aβ oligomer formation / dissociating the preformed Aβ oligomer	[107]
			Increased cell survival, antioxidative activity and decreasing AchE and BChE levels in Aβ-treated rat hippocampal cells	
			Inhibiting the Aβ-induced LPS and gene expression of TNF-α, IL-1β, COX-2, MIP-1α, MCP-1 and IP-10	[108]
			Suppressing the AChE activity and lipid peroxidation	[109]
	*Rehmannia glutinosa*		Improving scopolamine-induced cognitive impairment and cholinergic dysfunctions with decreasing TNF-α and IL-1β mRNA expression	[75]
			Increasing the gene expression of GDNF in astroglial cells via ERK1/2 and cPKC	[76]
		Catalpol	Increasing ChAT and BDNF levels	[73]
			Improvement of Aβ-induced memory and learning impariment via reducing Aβ and regulating ROS related enzymes	[74]
	*Citrus aurantium*		No direct reports regarding AD	
	*Pinellia ternata*		No direct reports regarding AD	
	*Wolfiporia extensa*		No direct reports regarding AD	

Aβ: amyloid beta, AβO: amyloid beta oligomers, AChE: acetylcholinesterase, AD: Alzheimer’s disease, ADAS: Alzheimer's disease assessment scale, Akt: protein kinase B (PKB), APP: amyloid precursor protein, BACE1: beta-site amyloid precursor protein cleaving enzyme 1, BChE: butyrylcholinesterase, BDNF: brain-derived neurotrophic factor, ChAT: choline acetyltransferase, COX-2: cyclooxygenase-2, cPKC: conventional protein kinase C, CREB: cAMP responsive element binding protein, ERK: extracellular signal-regulated kinases, GDNF: glial cell line-derived neurotrophic factor, GSK-3β: glycogen synthase kinase 3 beta, IL-1β: interleukin 1 beta, IP-10: interferon gamma-induced protein 10 (CXCL10), MAPK: mitogen-activated protein kinase, MCP-1: monocyte chemotactic protein 1(CCL2), MIP-1α: macrophage inflammatory protein 1-alpha (CCL3), MMSE: mini-mental state examination, PI3K: phosphoinositide 3-kinase, PS1: presenilin 1, PKA: protein kinase A, p-tau: phosphorylated tau, ROS: reactive oxygen species, TNF-α: tumor necrosis factor alpha.

#### a) In vivo and in vitro studies of JWWDT

The results of several *in vivo* and *in vitro* experiments suggest potential benefits of the use of JWWDT in the treatment of AD. Yabe, et al. [86] have demonstrated that the oral administration of JWWDT improves age-related memory disturbances in rats by increasing ChAT and NGF levels in the basal forebrain and frontoparietal cortex, respectively. Subsequently, Yabe, et al. ^(87)^ revealed that Polygalae Radix (PT root), a constituent of JWWDT, is involved in increasing the levels of ChAT activity in the basal forebrain and NGF secretion in astroglial cells. Similar to these results, an *in vitro* study of cultured basal forebrain and cerebral cortex cells from rat embryos has indicated that JWWDT treatment increases the levels of ChAT and NGF mRNA, respectively [88]. An animal study of the oral administration of JWWDT to mice with memory impairments induced by a thiamine-deficient diet has shown that JWWDT prolonged the shortened latency times in the PAT and shortened the transfer latency during the retention trial in the elevated plus-maze test, which indicated that learning and memory were improved. In addition to these behavioral assessments, immunohistochemical assays have shown that JWWDT attenuates the decrements in ChAT activity in the hippocampus [84]. Moreover, the results of these *in vivo* and *in vitro* animal studies support the results of clinical trials reporting cognitive-enhancing effects of JWWDT.

#### b) Clinical trials of JWWDT

Arai, et al. [89] have shown that the oral administration of JWWDT delayed the rate of cognitive decline on the MMSE of patients but did not change the amounts of Aβ_1-42_ and tau proteins in cerebrospinal fluids of AD patients. In another clinical study, the combined use of JWWDT and donepezil resulted in better clinical outcomes, including improved MMSE scores, Alzheimer's disease assessment scale (ADAS)-cognitive subscale scores, and improved CBF compared to donepezil monotherapy [90]. In short, the cognitive-enhancing effects of JWWDT appear to be very associated with increased cholinergic activity in the central nervous system (CNS) as well as improved CBF.

#### (2) Efficacy and mechanisms of action in AD pathology of each herb in JWWDT

Efficacy of single herbs of JWWDT is described on Table 4.

##### a) PN stalk

Methanol extracts of PN stalks have been reported to protect against neuronal cytotoxicity induced by Aβ_25-35_ in cultured rat cortical neurons by decreasing elevated levels of Ca^2+^, glutamate release and reactive oxygen species (ROS) generation [91].

##### b) CU peel

No studies have examined the effects of the CU peel on AD, however CU and its major component, nobiletin, have been reported to show neuroprotective effects against H_2_O_2_-induced oxidative stress in HT22 hippocampal neuronal cells via suppressing the phosphorylation of Jun N-terminal kinase, p38 and expression of apoptotic mediators such as bcl-2-like protein 4 (Bax) and caspase-3 [92].

##### c) GG root

The administration of aqueous GG root extract has been reported to result in spatial and memory enhancements scopolamine- and diazepam-induced amnesia in rats due to its anti-inflammatory and antioxidative properties [93, 94]. In addition, 2,2',4'-trihydroxychalcone derived from GG has been shown to mitigate memory impairments in an amyloid precursor protein (APP)-presenilin 1 (PS1) double-transgenic mouse model by reducing the production of Aβ through the specific and noncompetitive inhibition of BACE1 [95]. Furthermore, the oral administration of GG aqueous extract significantly reduced AChE activity even more than metrifonate, an irreversible AChE inhibitor, in the brains of Swiss albino mice [96].

##### d) PT root

Aqueous extracts of PT root have been reported to enhance axonal length and decrease the number of damaged neurons, but did not restore synaptic loss in Aβ_25-35_-treated rat cortical neurons [97]. Moreover, PT extract inhibited cell death induced by glutamate, Aβ and C-terminal fragment of APP in rat primary cultured neurons. In addition, PT extract improved scopolamine-induced cognitive impairment by dose-dependently and non-competitively inhibiting AChE activity [98].

##### e) SN root

Harpagoside, an iridoid glycoside purified from SN root, has been shown to alleviate the cognitive decline of rats induced by bilateral hippocampal injections of aggregated Aβ_1-40_. In addition, treatment with SN extracts attenuated the decreased number of ChAT-positive neurons and the decreased neurite outgrowth length induced by Aβ_1-42_ in ChAT-positive neurons in cultured primary cortical neurons. These effects appeared to be due to increased BDNF levels and the up-regulation of the MAPK/ phosphoinositide 3-kinase (PI3K) signaling pathway [99].

##### f) PG root

Large number of studies have been conducted to examine the effects of PG root on AD. Of the beneficial effects of PG root, reducing Aβ and hyperphosphorylated tau effects are considerable. Administration of PG root extract has been shown to reduce levels of Aβ_1-42_ and phosphorylated tau contents in AD model rats and promote cognitive function by mediating PI3K/Akt signaling pathway activity [100]. Moreover, ginsenoside Rg1, a saponin derived from PG, attenuated cognitive impairment in Tg mAPP mice by decreasing Aβ_1-40_, Aβ_1-42_ levels in the cerebral cortex and hippocampus. In addition, these modulating effects of APP process was mediated by decreased γ-secretase activity and increased protein kinase A/cAMP response element-binding (CREB) signaling pathway [101]. Remarkably, the administration of PG powder (4.5 g/day) for 12 weeks to patients with AD increased the patients’ Alzheimer’s disease assessment scale and MMSE scores and the scores decreased when PG was withdrawn [102].

##### g) ZJ fruit

No studies have reported direct evidence of the effects of ZJ fruit on AD. However, Snakin-Z, a peptide derived from ZJ fruit, has been reported to not only inhibit butyrylcholinesterase (BChE) and AChE but also to exhibit high antioxidative properties [103].

##### h) ZJ seed

Spinosin, a C-glycoside flavonoid derived from ZJ seeds, not only alleviated Aβ oligomer-induced cognitive decline but also inhibited the activation of microglia and ChAT [104]. In addition, spinosin improved memory impairments in mice with scopolamine-induced amnesia and enhanced cognitive performance by increasing adult hippocampal neurogenesis via increased phosphorylation of ERK, CREB and expression of mature BDNF in normal mice [105, 106].

##### i) ZO rhizome

The effects of ZO rhizome extracts on Aβ have been examined in *in vitro* studies. ZO extracts increased cell survival, antioxidative activity and decreased AChE and BChE levels in Aβ-treated primary adult rat hippocampal cells with high antioxidant activity. Moreover, ZO extracts inhibited Aβ oligomer formation and dissociated the preformed Aβ oligomers [107]. In THP-1 monocytes, ZO extract has been reported to inhibit lipopolysaccharide and gene expression of Aβ_1-42_-induced proinflammatory cytokines (TNF-α, IL-1β and cyclooxygenase-2 (COX-2)) and chemokines (MIP-1α (macrophage inflammatory protein 1-alpha, CCL3), MCP-1 (monocyte chemotactic protein 1, CCL2) and IP-10 (interferon gamma-induced protein 10, CXCL10)) [108]. In addition, aqueous extracts of ZO dose-dependently inhibited AChE activity and lipid peroxidation in the brains of rats [109].

### 4) Effects of DSS on AD

DSS (*Danggui shaoyao san* in Chinese, *Dangguijakyaksan* in Korean and Tokishakuyakusan in Japan) was first described in a traditional Chinese medical book (*Jin gui yao lue* ) published during the Han dynasty [110]. DSS has been used to treat ovarian dysfunctions, such as amenorrhea, luteal phase deficiency and anovulation [111, 112]. Moreover, previous studies have demonstrated that DSS has beneficial effects on middle-aged women with headaches, concomitant depression and sleep disturbances [113-115]. DSS is composed of the following six herbs: *Angelica sinensis* (AS) Oliv. Diels root, *Paeonia lactiflora* (PL) Pall. root, *Ligusticum chuanxiong* (LC) Hort rhizome, *Wolfiporia extensa* Peck Ginns sclerotium, *Atractylodes macrocephala* Koidz. rhizome and *Alisma orientalis* Sam. Juzep. rhizome [116-118]. In addition to the beneficial effects of DSS on menopause symptoms, the various effects of DSS in the CNS might be applicable in the treatment of AD.

#### a) In vivo and in vitro studies of DSS

In an animal model of dementia, DSS (100 and 200 mg/kg) ameliorated the disruptions in spatial memory induced by scopolamine administration [119]. Treatment of DSS (300 mg/kg) for 8 days ameliorated cognitive impairment by increasing cell survival in the hippocampus and mRNA levels of the GluR2 in rats with repeated cerebral ischemia [120]. Moreover, DSS (600 mg/kg) treatment for 14 days improved neurobehavioral performance by increasing adult subventricular neurogenesis and microvessel density in a middle cerebral artery occlusion-induced model of ischemic stroke. In addition, DSS activated the vascular endothelial growth factor and promoted endothelial nitric oxide synthase phosphorylation [117]. In addition to studies of the beneficial effects of DSS on cognitive function in AD, studies have examined the underlying mechanisms of these effects. DSS stimulates ChAT activity in the CNS. In ovariectomized mice used as a model of climacteric syndrome, the administration of DSS (*ad libitum* ) inhibited the decrease in ChAT activity in the cerebral cortex and dorsal hippocampus observed at 10 days after ovariectomy and improved the latency times on the PAT [121]. In addition, ChAT activities were increased in the ventral hippocampus by DSS treatment (500 mg/kg) for 1 month in menopausal rats [122].

However, after early increasing effects on ChAT activity, further treatment of DSS did not change ChAT activity in the hippocampus [122]. Similarly, DSS administration in an ovariectomized model did not change ChAT after early increasing effects on ChAT activity [121]. Even though the specifics of the mechanisms underlying the time-dependent responses to DSS are unclear, these discrepancies suggest that (1) the stimulating effects of DSS on ChAT may be limited and (2) another mechanism may be involved in the beneficial effects on cognitive function. In addition to the stimulating effects of DSS on ChAT, DSS improved cognitive abilities in step-down and MWM tests by increasing estradiol, nitric oxide and glycine levels in the hippocampus and blood plasma in a SAMP8 mouse AD model [116]. In addition, Toriizuka, et al. ^(121)^ have shown that DSS increased norepinephrine levels ay 20 days after ovariectomy. Furthermore, 7 days of treatment with DSS (100 μg) stimulate the neurite outgrowth of PC12 cells under NGF [69]. Based on the several effects of DSS on the CNS, it can be suggested that DSS is able to improve the symptoms of AD.

#### b) Clinical trials of DSS

Several studies examined the therapeutic effects of DSS on patients with AD. One case study reported that DSS treatment improved the scores of patients with AD on the Hasegawa dementia scale and reduced their behavioral symptoms [123]. DSS (7.5 g/day) administration for 8 weeks to patients with AD and mild cognitive impairment improved CBF and showed a tendency to improve the score of orientation to place in MMSE subscale [124]. In patients with mild cognitive impairments, 8 weeks of DSS treatment increased their MMSE scores and improved regional CBF [125], and 12 weeks of DSS administration improved the scores of Korean-Montreal Cognitive Assessment (K-MOCA) and MMSE as well [126].

#### (1) Efficacy and mechanisms of action in AD pathology of each herb in DSS

Efficacy of single herbs of DSS is described on Table 5.

##### a) AS root

AS root extract improved Aβ-induced memory impairments in rat models by inhibiting apoptosis, inflammation and nuclear factor κB (NF-κB) signaling pathway and the up-regulation of GDNF and BDNF in the hippocampus [127]. Furthermore, AS root extract inhibited Aβ-associated neurotoxicity in PC-12 cells and Neuro 2A cells via reducing oxidative stress through scavenging free radicals [128, 129]. The neuroprotective mechanisms underlying the inhibition of Aβ toxicity and tau phosphorylation by AS root treatment were related to regulation of PI3K/Akt/glycogen synthase kinase-3β (GSK-3β) signaling pathway [130]. Moreover, AS root extract exhibited anti-AChE effects (65.5%) in a study screening anti-AChE activities of 29 herbs [131].

##### b) PL root

Paeoniflorin, a monoterpene glycoside derived from water-soluble extracts of PL roots, inhibited inflammatory cytokines (TNF-α, IL-1β and IL-6) and chemokines (CXCL1 and CCL-2), NF-κB and VEGF/Flt-1 signaling pathways in Aβ_1-42_-treated rodent microglia [132]. In addition, paeoniflorin mitigated cognitive impairments through the regulation of suppressor of cytokine signaling 2 (SOCS2)/insulin receptor substrate-1 (IRS-1) pathway and activation of Akt and GSK-3β phosphorylation in rats with diabetes [133]. Moreover, lignans isolated from PL showed inhibitory effects on Aβ_1-42_ aggregation via hydrogen or non-hydrogen bond interaction with Aβ_1-42_ [134].

##### c) LC rhizome

Tetramethylpyrazine, a major alkaloid derived from LC rhizomes, inhibited proinflammatory mediators such as TNF-α, IL-1β, MCP-1 and ROS induced by interferon-γ and Aβ_25-35_ in primary microglial cells and organotypic hippocampal slice cultures [135].

**Table 5:** The efficacy and therapeutic mechanisms of single herbs constituting DSS on AD.

	Single herbs	Bioactive materials	Efficacy and mechanisms	Ref.
D S S	*Angelica Sinensis*		Improving Aβ-induced memory impairment by inhibiting inflammation and the NF-κB	[127]
			Upregulating GDNF and BDNF in the hippocampus	
			Inhibiting Aβ-associated neurotoxicity and Aβ aggregation by reducing of oxidative stress	[128]
				[120]
			Neuroprotection to Aβ toxicity and tau phosphorylation through regulating PI3K/Akt/GSK-3β signaling pathway	[130]
			Decreasing AChE activity	[131]
	*Paeonia lactiflora*			
		Paeoniflorin	Attenuating inflammation induced by Aβ_1-42_ in rodent microglia via inhibiting inflammatory cytokines (TNF-α, IL-1β and IL-6) and chemokines (CXCL1 and CCL-2)	[132]
			Inhibiting the NF-κB and VEGF/Flt-1 signaling pathways	
			Mitigating cognitive impairment via regulating SOCS2/IRS-1 in rats with diabetic	[133]
			Promoting phosphorylation of Akt and GSK-3β	
		Lignans	Preventing the Aβ_1-42_ aggregation via hydrogen or non-hydrogen bond interaction with Aβ_1-42_	[134]
	*Ligusticum chuanxiong*			
		Tetramethylpyrazine	Inhibiting pro-inflammatory mediators (TNF-α, IL-1β, MCP-1 and ROS) induced by interferon-γ and Aβ_25-35_	[135]
	*Alisma orientale*		No direct reports regarding AD	
	*Atractylodes macrocephala*		No direct reports regarding AD	
	*Wolfiporia extensa*		No direct reports regarding AD	

AD: Alzheimer’s disease, Aβ: amyloid beta, Akt: protein kinase B (PKB), AChE: acetylcholinesterase BDNF: brain-derived neurotrophic factor, CXCL1: C-X-C motif chemokine ligand 1, CCL-2: C-C motif chemokine ligand 2, Flt-1: functions of the VEGF receptor-1, GDNF: glial cell line-derived neurotrophic factor, Glu: glutamic acid, GSK-3β: glycogen synthase kinase 3 beta, His: histidine, IL-1β: interleukin 1 beta, IL-6: interleukin 6, IRS-1: insulin receptor substrate-1, NF-κB: nuclear factor kappa-light-chain-enhancer of activated B cells, PI3K: phosphoinositide 3-kinase, SOCS2: suppressor of cytokine signaling 2, TNF-α: tumor necrosis factor alpha, VGEF: vascular endothelial growth factor.

### 5. Effects of HLJDT on AD

HLJDT (*Huanglian jiedu tang* in Chinese, *Hwanglyeonhaedoktang* in Korean and *Orengedokuto* in Japan) was first described in a traditional Chinese medical book (*Wai tai mi yao* ) published during the Tang dynasty (752 AD). HLJDT has historically been used to treat excessive body heat, nausea and insomnia [136, 137]. This formula can be used to treat various clinical symptoms, such as gastric mucosal lesions [138, 139], rheumatoid arthritis [140-142], dermatitis [143] and inflammatory diseases [144, 145]. HLJDT consists of the following 4 herbs: *Coptis chinensis* (CC) Franch. rhizome, *Scutellaria baicalensis* (SB) Georgi root, *Phellodendron amurense* (PA) Rupr. cortex and *Gardenia jasminoides* (GJ) Ellis fruit [146]. The mechanisms underlying the anti-dementia and cognitive-enhancing effects of HLJDT vary.

#### a) In vivo and in vitro studies of HLJDT

A morphologic and histopathologic study have shown that administration of HLJDT decreased damages of hippocampal neurons and formation of senile plaque with increased levels of superoxide dismutase and decreased levels of malondiadehyde [147]. Moreover, berberine, an isoquinoline alkaloid derived from CC, improved cognitive functions by suppressing Aβ accumulation, microglial activation and Akt/GSK3 signaling pathway [148]. In contrast, Durairajan, et al. [149] have reported that HLJDT treatment increases the amyloidogenic metabolism of APP and production of Aβ in N2a-SwedAPP cells, which are used as an *in vitro* model of AD. They also showed anti-APP and Aβ-production effects of a modified formula of HLJDT (removed SB root from original formula). In addition, HLJDT has cognitive enhancing effects in animal models. Similar to the results obtained with donepezil administration, coptisine (50 mg/kg/day for 1 month), an active constituent of HLJDT also significantly improved the spatial memory impairments by reducing Aβ_1-42_ accumulation and neuronal damage in APP/PS1 transgenic mice [150]. Moreover, effects of HLJDT on cerebral ischemia suggest that HLJDT can be used to treat AD to improve cognitive dysfunction. The oral administration of HLJDT ameliorated the disruption of spatial memory induced by cerebral ischemia in rats and mice [119, 151]. The memory-enhancing effects of HLJDT are mediated by inhibiting the decrement of ACh levels in the cerebral cortex, hippocampus and striatum [151]. More on ACh increasing effects, HLJDT inhibited indoleamine 2,3-dioxygenase (IDO), a key enzyme in kynurenine pathway, *in vitro* [152]. Since over-activation of kynurenine pathway is thought to be involved in AD pathogenesis, the IDO-inhibiting effects of HLJDT might be one strategy for development AD treatment.

#### b) Clinical trials of HLJDT

A recent clinical trial has reported that 12 weeks of treatment with HLJDT has therapeutic potential in patients with AD treated with pitavastatin. Patients treated with pitavastatin and HLJDT show significantly lower levels of Aβ, phosphorylated tau and inflammatory factors [153]. A case study has reported that supplementary treatment of HLJDT with YGS decreased BPSD in the patient with AD exhibiting aggressive behaviors [136]. Overall, the results of the clinical and nonclinical studies have suggested that HLJDT has Aβ-reducing effects and ameliorates the symptoms of AD.

#### (1) Efficacy and mechanisms of action in AD pathology of each herb in HLJDT

Efficacy of single herbs of HLJDT is described on Table 6.

##### a) CC rhizome

Coptisine, an active constituent of HLJDT, reduced Aβ formation [150]. Through the Aβ-reducing mechanisms of coptisine, the expression of IDO, which is induced by Aβ, was suppressed by HLJDT and its main constituents *in vivo* and *in vitro* [150, 152]. Similar findings have been observed in studies examining the effects of CC rhizome on AD-related pathogenesis. In that study, six alkaloids (berberine, palmatine, jatrorrhizine, coptisine, groenlandicine and epiberberine) isolated from CC rhizome shown to have inhibitory effect on AChE, BChE and two alkaloids (groenlandicine and epiberberine) exhibited inhibitory effects on BACE1. Moreover, jatrorrhizine, groenlandicine and coptisine have antioxidant effects [154].

##### b) SB root

Baicalein (5,6,7-trihydroxyflavone), a major flavonoid isolated from SB root, improved learning and spatial memory by activating γ-aminobutyric acid (GABA) type A receptors and inhibited Aβ production through increasing nonamyloidogenic processing of APP in Tg2576 mice [155]. An additional notable effect of baicalein was hippocampal neurogenesis promoting effect. Baicalein treatment ameliorated cognitive impairment induced by irradiation via increased hippocampal neurogenesis and up-regulated BDNF-pCREB signaling pathway [156]. Another major active component, wogonin, has been reported to improve impaired cognitive function in 3xTg-AD mice and reduce the levels of β-secretase, Aβ aggregation and phosphorylated tau in AD cells (Tet-On Aβ_42_-GFP SH-SY5Y neuroblastoma cells). In addition, wogonin also protected against apoptosis and increased mitochondrial membrane potential via suppressing the cleaved poly (ADP-ribose) polymerase (PARP) and expression of Bax [157].

##### c) PA cortex

Ethanol extracts of PA cortex have been reported to have protective properties against Aβ-induced neurotoxicity via up-regulating the B-cell lymphoma-2 (Bcl-2)/Bax expression ratio and down-regulating the release of cytochrome c and caspase-3 expression in PC12 cells [158].

##### d) GJ fruit

GJ fruit extracts have reduced Aβ-induced cytotoxicity through the reduction of oxidative stress in PC12 cells [159]. Moreover, of the iridoides glucosides isolated from GJ fruit, 6'-O-trans-p-coumaroylgeniposide and 6'-O-acetylgeniposide showed the short-term memory-enhancing effects in an Aβ transgenic drosophila model [160]. In addition, geniposide isolated from GJ inhibited Aβ_1-42_-induced cholinergic deficits through the inhibition of AChE activity and up-regulation of ChAT in primary hippocampal neurons. Moreover, geniposide inhibited over-activated p38 MAPK, ERK1/2 and receptors for advanced glycation end products (RAGE) which is mediator of Aβ related neurotoxicity, thereby improving cholinergic defects and amyloidosis as well as mitigate cognitive impairments in APP/PS1 mice [161].

**Table 6:** The efficacy and therapeutic mechanisms of single herbs constituting HLJDT on AD.

	Single herbs	Bioactive materials	Efficacy and mechanisms	Ref.
H L J D T	*Coptis chinensis*			
		*Coptisine*	Suppressing IDO expression via reducing Aβ formation	[150, 152]
			Inhibiting AChE and BChE activity	[154]
		*Berberine*	Inhibiting AChE and BChE activity	[154]
		*Palmatine*		
		*Jatrorrhizine*		
		*Groenlandicine*	Inhibiting AChE, BChE and BACE1 activity	
		*Epiberberin*		
	*Scutellaria baicalensis*			
		Baicalein	Improving AD-like pathology together with improved cognitive performance by activation of the GABA type A receptors and the α-secretase processing of APP.	[155]
			Alleviating spatial learning and memory deficits induced by gamma-ray radiation.	[156]
			Stimulating neurogenesis and up-regulating BDNF-pCREB pathway	
		Wogonin	Improving impaired cognitive function in 3xTg AD mcie	[157]
			Reducing β-secretase levels, Aβ aggregation and phosphorylated tau	
			Protecting against apoptosis and mitochondrial membrane potential elevation via suppression of the cleaved PARP and expression of Bax	
	*Phellodendron amurense*		Protecting Aβ-induced neurotoxicity via upregulating Bcl-2/Bax ratio and down-regulating cytochrome c and caspase-3 expression	[158]
	*Gardenia jasminoides*		Reducing Aβ-induced cytotoxicity via reduction of oxidative stress	[159]
		6'-O-trans-p-coumaroylgeniposide	Enhancing short-term memory in Aβ transgenic drosophila model	[160]
		6'-O-acetylgeniposide		
		Geniposide	Inhibiting the activity of AChE and increasing the activity of ChAT	[161]
			Mitigating the cognitive impairment in APP/PS1 mice by inhibition of Aβ_1-42_-induced cholinergic deficit and amyloidosis via inhibition of MAPK	

AD: Alzheimer’s disease, Aβ: amyloid beta, AChE: acetylcholinesterase, APP: amyloid precursor protein, Bax: bcl-2-like protein 4, Bcl-2: B-cell lymphoma-2, BDNF: brain-derived neurotrophic factor, ChAT: choline acetyltransferase, GABA: gamma-aminobutyric acid, IDO: indoleamine 2,3-dioxygenase, MAPK: mitogen-activated protein kinase, PARP: poly (ADP-ribose) polymerase, pCREB: phosphorylation of cAMP response element-binding protein, 3xTg: three mutations associated with familial Alzheimer's disease (APP Swedish, MAPT P301L, and PSEN1 M146V).

## 6. Discussion

AD is a disease that threatens the quality of life of the patients with the disease as well as their caregivers [162-164]. Moreover, the global prevalence of AD has been persistently increasing and is expected to continue to grow [165]. Therefore, several medicinal interventions have been developed and evaluated. Nonetheless, only a few FDA-approved drugs are in use in most clinical settings. Meanwhile, Oriental herbal medicine, especially herbal formulae, has long been used to treat the symptoms of amnesia and dementias, including AD, in East Asian countries. However, the effects of Oriental herbal medicine on the symptoms of AD have been scientifically investigated for only a few decades. Moreover, TOMs are not widely adopted in Western medicine nor has it been studied by laboratory experiments before the last few decades. Thus, we focused on five herbal formulae that are used frequently in clinical practice.

One of the most notable effects of the formulae is inhibition of Aβ accumulation. As reviewed, several studies regarding formulae and their constituents have reported the inhibitory effects on Aβ aggregation. This might provide an important implication to potential treatments for AD. With regard to aforementioned formulae or single herbs, recent studies have revealed beneficial effects of TOMs on AD. One recent study has examined that the combination of herbal formula named GRAPE (mainly consisted with *Panax ginseng* , *Rehmannia glutinosa* , *Acorus tatarinowii* , *Polygala tenuifolia* , *Epimedium brevicornu* ) with conventional therapy such as donepezil and/or memantine was more effective on cognitive function in AD patients than conventional therapy alone [166]. Another recent *in vivo* studies have shown that *Bushen Tiansui* decoction (consisted with *Epimedium brevicornum Maxim* , *Polygonum multiflorum Thunb* , Chinemys reevesii, Fossilia Ossis Mastodi, *Polygala* , *Acorus tatarinowii* ) and triterpenoid saponins of *Xanthoceras sorbifolia* Bunge decreased hippocampal damages and cognitive deficits induced by Aβ aggregation [167, 168]. Moreover, an *in vitro* study reported that extracts from the leaves of *Cassia tora* Linn. could suppress the Aβ aggregation [169].

However, clinical application of TOMs should be conducted after deliberate consideration of several aspects. In order to apply TOMs in clinical use, TOMs should ensure their safety. Although, TOMs have long been used, there were concerns regarding toxicity induced by heavy metals, pesticides and microorganisms [170]. Another aspect should be considered is that TOMs have established by a unique principle in herbal medicinal prescription as well [37, 171]. For instance, formulae that prescribed in TOMs is made with several herbs under the consideration of the effects of each active ingredient and principally consisted with major herb (*Jun* , emperor or monarch), complementary herb (*chen* , minister), neutralizing herb (*zuo* , assistant) and delivery herb (*shi* , servant or guide) [172, 173]. Therefore, evaluative studies on TOMs’ safety as well as pharmacokinetics and pharmacodynamics of formulae should be conducted for clinical use of TOMs.

The limitation of present review is that this study might not fully ensure the internal and external validity, since this review was not conducted with systematic methodology. Nonetheless of the limitation, reviewing the efficacy and mechanisms of action of standardized TOM formulae commonly used in the clinical practice for patients with cognitive impairments and related symptoms could provide noteworthy implications to potential therapies for AD.

## 7. Conclusion

The major conclusions of this review are the following. First, traditional Oriental herbal medicine is under evaluated despite the extended periods of its use. Recent advances in high-performance liquid chromatography might provide higher peak efficiencies that can be used to more specifically identify each ingredient and component of the formulae. Second, although the inhibitory effects of these herbal formulae on Aβ accumulation are still controversial, the underlying mechanisms of the inhibitory effects of the herbal formulae (e.g., YGS and HLJDT) and/or bioactive components need to be examined further.
